# Intrinsic coupling between spatially-separated surface Fermi-arcs in Weyl orbit quantum Hall states

**DOI:** 10.1038/s41467-021-22904-8

**Published:** 2021-05-06

**Authors:** Shinichi Nishihaya, Masaki Uchida, Yusuke Nakazawa, Markus Kriener, Yasujiro Taguchi, Masashi Kawasaki

**Affiliations:** 1 Department of Applied Physics and Quantum-Phase Electronics Center (QPEC), University of Tokyo, Tokyo, Japan; 2 Precursory Research for Embryonic Science and Technology (PRESTO), Japan Science and Technology Agency (JST), Tokyo, Japan; 3 Department of Physics, Tokyo Institute of Technology, Tokyo, Japan; 4 RIKEN Center for Emergent Matter Science (CEMS), Wako, Japan

**Keywords:** Quantum Hall, Surfaces, interfaces and thin films, Topological insulators

## Abstract

Topological semimetals hosting bulk Weyl points and surface Fermi-arc states are expected to realize unconventional Weyl orbits, which interconnect two surface Fermi-arc states on opposite sample surfaces under magnetic fields. While the presence of Weyl orbits has been proposed to play a vital role in recent observations of the quantum Hall effect even in three-dimensional topological semimetals, actual spatial distribution of the quantized surface transport has been experimentally elusive. Here, we demonstrate intrinsic coupling between two spatially-separated surface states in the Weyl orbits by measuring a dual-gate device of a Dirac semimetal film. Independent scans of top- and back-gate voltages reveal concomitant modulation of doubly-degenerate quantum Hall states, which is not possible in conventional surface orbits as in topological insulators. Our results evidencing the unique spatial distribution of Weyl orbits provide new opportunities for controlling the novel quantized transport by various means such as external fields and interface engineering.

## Abstract (web summary)

The spatial distribution of the quantized transport due to the presence of Weyl orbits in topological semimetals remains elusive. Here, the authors report concomitant modulation of doubly-degenerate quantum Hall states, evidencing intrinsic coupling between two spatially separated surface states in the Weyl orbits of a Dirac semimetal film.

## Introduction

Topological phases of matter hosting nontrivial surface states have enriched our understanding of transport phenomena^1–3^. In contrast to topological insulators (TIs), where the surface transport can be isolated from the bulk one by tuning the Fermi level (*E*_F_) within the bulk energy gap^1,2^, topological Dirac and Weyl semimetals (DSM/WSM) have a characteristic feature that the gapless bulk and surface states merge at Weyl nodes^3^, leading to unique interplay between them^4–7^. One of the consequences is the formation of Weyl orbit under magnetic fields, which weaves together two spatially separated surface Fermi-arc states via the bulk nodes^4,5^. Owing to the completion of a closed cyclotron motion by the two orbital segments on the opposite surfaces, the Weyl orbit has been predicted to form Landau levels, leading to two-dimensional (2D) quantum transport^4,5,8^.

**Fig. 1:**
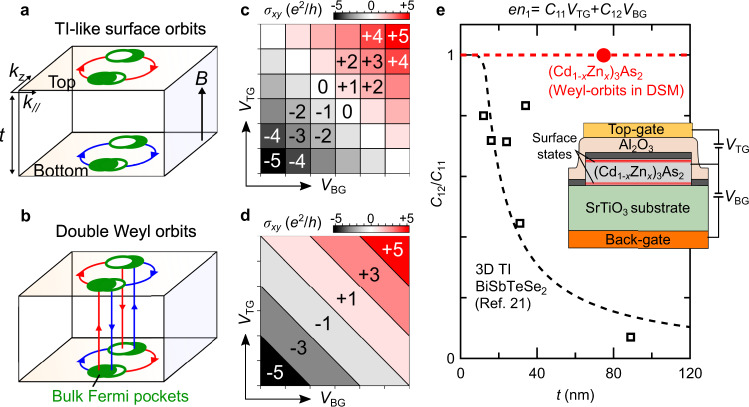
Quantum Hall signature of topological surface orbits. **a** Conventional topological-insulator-like (TI-like) surface orbits independently localized on each film surface. **b** A pair of Weyl orbits connecting the top and bottom surface states via the bulk chiral modes. **c**, **d** Expected appearance pattern of quantum Hall states for TI-like surface orbits (**c**) and for doubly degenerate Weyl orbits (**d**) in a Dirac semimetal film. **e** Capacitive coupling of carrier density for each surface orbit (*n*_1_, *n*_2_) to the top and back-gate voltages (*V*_TG_ and *V*_BG_) as defined in Eq. (3). While the two surface orbits in the TI-like case are decoupled and *C*_12_/*C*_11_ decreases to 0 as the film thickness *t* increases^21^ (black dashed line), the carrier density of the paired Weyl orbits is expected to be modulated simultaneously by both *V*_TG_ and *V*_BG_ (*C*_12_/*C*_11_ = 1) independent of the film thickness (red dashed line). The inset is a schematic illustration of the dual-gate configuration fabricated on the (Cd_1−*x*_Zn_*x*_)_3_As_2_ film, where electrons are depleted when *V*_TG_ (*V*_BG_) < 0.

Experimentally, transport signatures of the 2D surface states have been reported by several groups^9–14^, mainly for the typical DSM material Cd_3_As_2_. Distinct from the 2D bulk transport triggered by sub-band splitting under confinement^15,16^, the 2D surface transport is typically accompanied by coexisting 3D bulk state, and is characterized by a larger Fermi surface and band mass than their bulk counterparts^10–14^. It exhibits not only quantum oscillations, but also quantum Hall (QH) effect in high magnetic fields or at low carrier concentrations^12–14^, attracting growing attention as novel quantized transport in a 3D system. On the other hand, an important question still under debate is whether the two opposite surface states are coupled through the bulk nodes in the quantized state. In particular, DSM with two Fermi-arcs on the same surface may also host TI-like surface orbits independently localized on the opposite surfaces^17,18^ (Fig. 1a), in contrast to the Weyl orbits (Fig. 1b).

A Weyl orbit has two features distinct from the TI-like conventional orbit. One is the bulk tunneling process via the 1D bulk chiral mode formed parallel to the magnetic field. As proposed based on a semiclassical picture, the bulk tunneling process may be experimentally detectable as phase shift of QH plateaus, when the bulk thickness is modulated^4,5,12^. It should be noted, though, that a similar plateau shift can be induced also by inhomogeniety of sheet carrier density, which varies proportionally to the thickness^13,14^. Therefore, it is challenging to verify the existence of Weyl orbits, excluding extrinsic origins.

Here, we take a different approach by focusing on another feature of the Weyl orbit, namely its unique spatial distribution extending over two distanced sample surfaces. The key observation to distinguish the Weyl orbit from the TI-like orbit is the response of surface transport to electric fields applied in a dual-gate device configuration. In the TI-like conventional case (Fig. 1a), each of the two surfaces independently hosts a 2D electronic state^19–21^. The total QH filling factor *ν* can be expressed by simple summation of those of the two surfaces (*ν*_1_ and *ν*_2_),

1 $$\nu =\left({\nu }_{1}+\frac{1}{2}\right)+\left({\nu }_{2}+\frac{1}{2}\right)=0,\pm \!\!1,\pm \!\!2\cdots \ .$$

The field effect applied on each surface modulates *ν*_1_ or *ν*_2_ only independently, thus giving a checkerboard QH plateau pattern of *ν* as depicted in Fig. 1c^21^. In the Weyl orbit case (Fig. 1b), on the other hand, the two Weyl orbits with opposite chirality are formed across the top and bottom surfaces, and the electron density of both orbits can be modulated simultaneously by top- and/or back-gating. If the two Weyl orbits are perfectly degenerate in the DSM phase, the total *ν* is given by

2 $$\nu =\left({\nu }_{1}+\frac{1}{2}\right)+\left({\nu }_{2}+\frac{1}{2}\right)=2\left({\nu }_{1}+\frac{1}{2}\right)=\pm \!\!1,\pm \!\!3,\pm \!\!5\cdots \ ,$$

resulting in a stripe pattern extending along the constant sheet density line as shown in Fig. 1d. Here, we assume the half-integer type QH effect for the Weyl orbits. This is because the surface dispersion of the two Fermi-arcs of DSM is expected to possess a single Dirac point at the Brillouin zone center, owing to the nontrivial *Z*_2_ invariant as in TIs^17,18^.

The difference in the appearance pattern of the QH states can be also described by the difference in the capacitive coupling of the surface carrier densities to the two gate voltages as presented in Fig. 1e. Denoting the carrier densities of the two surface orbits as *n*_1_ and *n*_2_, and top- and back-gate voltages as *V*_TG_ and *V*_BG_, their coupling relation is simply expressed by a 2 × 2 capacitance *C* matrix as follows,

3 $$\left(\begin{array}{l}e{n}_{1}\\ e{n}_{2}\end{array}\right)=\left(\begin{array}{ll}{C}_{11}&{C}_{12}\\ {C}_{21}&{C}_{22}\end{array}\right)\left(\begin{array}{l}{V}_{{\rm{TG}}}\\ {V}_{{\rm{BG}}}\end{array}\right).$$

Here, *e* is the elementary charge. Since the carrier densities of the two Weyl orbits can be modulated by both *V*_TG_ and *V*_BG_, the off-diagonal term ideally equals to the diagonal term (*C*_12_/*C*_11_ = 1) independent of the film thickness (Fig. 1e). This is different from the TI case, where the two surface orbits are perfectly isolated (*C*_12_/*C*_11_ = 0) by the insulating bulk state in the three-dimensionally thick limit^21^. We note that reducing the film thickness in the TI case leads to finite coupling between the two surfaces (*C*_12_/*C*_11_ ≠ 0). Even in such a case, the checkerboard pattern of the QH states (Fig. 1c) only deforms from a tetragonal to a diamond pattern^21^, and is still distinct from the stripe pattern in the Weyl orbit case (Fig. 1d).

In this report, we demonstrate that the surface QH states in a 75 nm thick DSM (Cd_1−*x*_Zn_*x*_)_3_As_2_ film exhibit a clear stripe pattern in dual-gate *V*_TG_–*V*_BG_ scans. Our results indicate the intrinsic coupling of the two surfaces states even in a thick enough region, conclusively evidencing the unique distribution of the Weyl orbits extending across the entire film.

## Results

### Sample and quantum transport properties

**Fig. 2:**
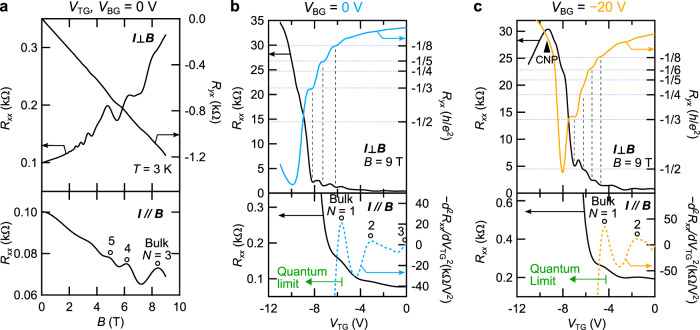
Quantum transport of a 75 nm thick (Cd_1−*x*_Zn_*x*_)_3_As_2_ film. **a** Field dependence of resistance *R*_*xx*_ measured in transverse field (*I* ⊥ *B*, upper panel) and longitudinal field (*I* ∥ *B*, lower panel) configuration at *V*_TG_,*V*_BG_ = 0 V. The right axis shows Hall resistance *R*_*yx*_. **b**, **c** *V*_TG_ scans of *R*_*xx*_ (upper panel for *I* ⊥ *B*, lower panel for *I* ∥ *B*) and *R*_*yx*_ measured at *B* = 9 T with *V*_BG_ set to 0 V (**b**) and −20 V (**c**). To clearly present the bulk oscillations, the second derivatives of *R*_*xx*_ with respect to *V*_TG_ are also shown on the right axis of the lower panels. Quantum Hall plateaus clearly develop as the bulk state reaches the quantum limit. A carrier type inversion is induced as the film is depleted across the charge neutrality point (CNP) in **c**.

Zn-doped Cd_3_As_2_ thin film was fabricated on a single-crystalline SrTiO_3_ (100) substrate by the combination of pulsed laser deposition and solid-phase epitaxy, following the same procedure described in previous papers^15,22,23^. The film is oriented in the [112] crystallographic direction of Cd_3_As_2_, and the Fermi-arc states on the film surfaces have finite length connecting between the projections of the bulk nodes residing on the [001] axis. Zn-doping is for reducing the residual electron density of Cd_3_As_2_, and the doping concentration (*x* = 0.07) is designed to be low enough to maintain the nontrivial DSM phase (*x* < 0.2)^14^. Figure 2a summarizes the magneto-transport of a 75 nm thick film measured at *V*_TG_, *V*_BG_ = 0 V and temperature *T* = 3 K. Quantum oscillations in the resistance *R*_*xx*_ are clearly observed in both transverse (*I* ⊥ *B*) and longitudinal (*I* ∥ *B*) field configurations, which ensures that the film is thick enough to keep the 3D bulk state with gapless bulk nodes required for the formation of the Weyl orbit. At the electron density of 6 × 10^17^ cm^−3^, the conduction is dominated mainly by the bulk transport, and the signature of the surface transport is barely observed without gating.

Once depleting the electron density by gating, on the other hand, the surface transport and its evolution into QH states become more evident. Figure 2b, c presents *V*_TG_ scans of *R*_*xx*_ and Hall resistance *R*_*yx*_ at 9 T with *V*_BG_ set to 0 and −20 V (see also Supplementary Fig. 1). In the lower panels, we also show *R*_*xx*_ curves measured with *I* ∥ *B* configuration and their second derivative with respect to *V*_TG_. Plateau structures in *R*_*yx*_ accompanied by sharp drops of *R*_*xx*_ start to appear as the occupation of the bulk state is depleted to the quantum limit (see also Supplementary Fig. 3). When depleted by both *V*_TG_ and *V*_BG_, the system crosses the charge neutrality point, changing the majority carrier type from *n*- to *p*-type (Supplementary Fig. 2).

It is worth mentioning that even with bulk occupation, the QH plateaus with integer filling factors appear at the fields determined by *B* = *n*_2D_*h*/*eν* with the total sheet carrier density *n*_2D_, the elementary charge *e*, and the Planck constant *h*, as in a conventional 2D system. This feature indicates the unconventional involvement of bulk carriers in the formation of QH states, which may be behind the realization of quantized conduction even in the 3D system. It has been also discussed recently that the flatness of the Landau levels formed by the interacting bulk and surface states leads to a significant probability of the electron transport taking place at the sample boundary, enabling the quantization similar to a 2D system^24^. In this sense, the conventional picture of parallel surface and bulk conduction established for TIs is not applicable here, because in such a case, the presence of bulk occupation would make the QH plateaus deviate from integer values and suppress the quantization^20^.

### Dual-gate mapping of surface quantum Hall effect

**Fig. 3:**
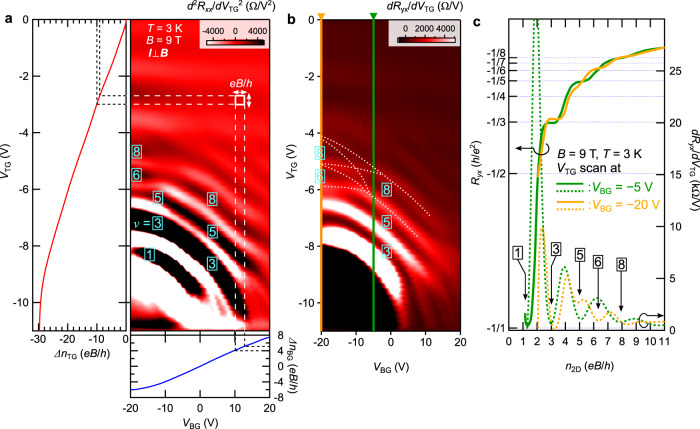
Dual-gate modulation of the surface quantum Hall states. **a**, **b** Mappings of second derivative of resistance *R*_*xx*_ (**a**) and first derivative of Hall resistance *R*_*yx*_ (**b**) as a function of *V*_TG_ and *V*_BG_. The modulation of the sheet electron densities (Δ*n*_TG_, Δ*n*_BG_) in the *V*_TG_ and *V*_TG_ scans are also shown in **a**. The quantum Hall state of each filling factor *ν* exhibits a stripe pattern over a wide range of carrier density, indicating the concomitant modulation of the paired Weyl orbits by both the top- and back-gating. Overlaid dashed lines in **b** show the fine structure of the Landau level crossings caused by the degeneracy lifting of the two Weyl orbits. **c** *V*_TG_ scans of *R*_*yx*_ and its first derivative (along the vertical lines at *V*_BG_ = −5 and −20 V shown in **b** plotted as a function of sheet electron density *n*_2D_. The *n*_2D_ axis is presented in units of the Landau level degeneracy *eB*/*h*.

Next, we present *V*_TG_–*V*_BG_ mappings of the surface QH states measured at 9 T in Fig. 3. To explicitly show the oscillations in *R*_*xx*_ and plateau transitions in *R*_*yx*_, we plot the second derivative of *R*_*xx*_ in Fig. 3a and the first derivative of *R*_*yx*_ in Fig. 3b. The most striking feature of the mappings is that the *R*_*xx*_ minima and *R*_*yx*_ plateaus of the QH states (corresponding to darker regions in Fig. 3a, b) extend over a wide range of *V*_TG_ and *V*_BG_ in a stripe pattern as shown in Fig. 1d. Moreover, the filling factor *ν* does not take every integer, but shows double degeneracy such as in the *ν* = 1, 3, 5 states. These results clearly indicate the presence of a pair of Weyl orbits. Each Weyl orbit has orbital segments on the top and bottom film surfaces so that both *V*_TG_ and *V*_BG_ can concomitantly modulate the surface QH states. Importantly, the *V*_TG_ and *V*_BG_ scans cover a sufficiently wide carrier density range (>10 *eB*/*h* at 9 T), allowing to distinguish the checkerboard (Fig. 1c) and the stripe (Fig. 1d) patterns. We also note that the finite curvature of the stripe patterns observed in Fig. 3a, b are caused by the saturating behavior of the gate-modulated carrier density around higher gate voltages.

In addition to the unique coupling between the opposite surface states, the appearance of QH filling factors further reveal sensitive responses of the Weyl orbits to the external electric and magnetic fields, reflecting their degeneracy lifting induced by the topological phase transition from DSM to WSM. In DSM, the application of external electric fields *E* (magnetic fields *B*) results in splitting of the bulk Dirac node into a pair of Weyl nodes with opposite chirality by breaking the inversion (time-reversal) symmetry^25–27^. In both the *E*-driven and *B*-driven cases, a pair of Weyl nodes shifts oppositely both in the energy and momentum directions, depending on the underlying band structure^25,27^. The direct consequence of these bulk node splitting effects is the degeneracy lifting of the two Weyl orbits with opposite chirality. The Landau levels of the two Weyl orbits split and cross with each other, depending on the magnetic and electric fields, leading to deviations from the doubly degenerate pattern shown in Fig. 1d.

### Degeneracy lifting of Weyl orbits in Dirac semimetal

Experimentally, the effects of *E*-driven and *B*-driven splitting can be confirmed separately in some scans. For example, in the region between the *V*_TG_ scans at *V*_BG_ = −5 and −20 V in Fig. 3a, b, one can notice that there are several level crossings (denoted by the white dashed lines in Fig. 3b), which result in additional plateau transitions. Figure 3c compares the typical *V*_TG_ scans of *R*_*yx*_ and its first derivative at *V*_BG_ = −5 and −20 V as a function of sheet density *n*_2D_. Not only a series of odd integer *ν*, but also even integer *ν* such as *ν* = 6 and 8 can be observed, indicating the lifting of the double degeneracy. These results obtained by scanning the gate voltages at a fixed magnetic field can be interpreted as the manifestation of the *E*-driven splitting effect.

**Fig. 4:**
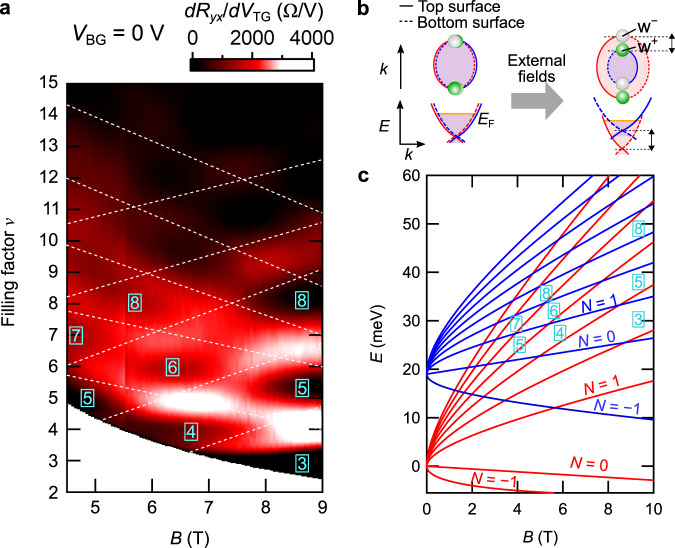
Complex plateau transitions induced by external fields. **a** Mapping of the first derivative of the Hall resistance *R*_*yx*_ as a function of magnetic field *B* and filling factor *ν*. The mapping is converted from the original *B*–*n*_2D_ mapping data via *ν* = *n*_2D_*h*/*eB* (see also Supplementary Figs. 4 and 5). Plateau transitions between the quantum Hall states (corresponding to the brighter regions in the mapping) indicate the existence of multiple crossings of the Landau levels. The overlaid dashed lines are guides for eyes. **b** Schematic illustrations for the external-field-induced splitting of the Weyl nodes of opposite chirality (W^±^) and consequent degeneracy lifting of the two Weyl orbits. **c** Fan diagram with complex Landau level crossings simulated by introducing an effective Zeeman splitting, and a relative energy shift between the two Weyl orbits (see Supplementary Note 5).

On the other hand, the *B*-driven splitting of the Weyl orbits is observed by scanning the magnetic field. We present in Fig. 4a the mapping of the QH states as a function of magnetic field *B* and filling factor *ν*. It is evident that complex level crossings take place, resulting in the alternate appearance of odd and even integer *ν*, depending on the magnetic field. The external-field-induced bulk node splitting in the energy and momentum directions is reflected in the asymmetric modulation of the surface band dispersion of opposite chirality, including its energy position, Fermi surface area, and Fermi velocity (Fig. 4b). Taking these effects into account, we simulate the situation observed in Fig. 4a by assuming the *B*-driven splitting effect appearing as an effective Zeeman term and the *E*-driven splitting effect as relative energy offset between the two Weyl orbits (see Supplementary Note 5 for details of the model). Choosing suitable parameters, the simulated fan diagram shown in Fig. 4c exhibits multiple Landau level crossings, and well accounts for the complex plateau transitions seen in Fig. 4a. We have also confirmed that the level crossing pattern in Fig. 4a changes, when measured with different *V*_BG_, indicating the *E*-driven splitting of the two Weyl orbits in the gate configuration (see Supplementary Fig. 6).

## Discussion

In previous studies of the surface QH states^10,12–14^, observed QH filling factors and degeneracy greatly differ by experimental setups. This variation has been hindering the consistent understanding of the quantized transport. At this point, our mapping results in Figs. 3 and 4 indicate that the field-induced splitting of the paired Weyl orbits in DSM plays a critical role in determining the detailed appearance of the QH filling factors. We therefore suggest that the Weyl orbit degeneracy lifting sensitive to external fields may also explain the variation in the previous observations^10,12–14^.

In summary, we have systematically investigated the surface QH states in a dual-gated DSM film. Our results provide transport evidence of an intrinsic coupling between the two spatially separated surface states in the presence of Weyl orbits. The entire mappings of the QH states as functions of gate voltages and magnetic field further reveal the complex appearance of the filling factor, reflecting the degeneracy lifting of the paired Weyl orbits by external magnetic and electric fields. Considering that the unique spatial distribution of the Weyl orbit is implemented by electron tunneling via the bulk chiral mode parallel to the magnetic field, it would be interesting to further investigate how the connectivity between the two Fermi-arcs on the opposite surfaces can be modulated, such as by applying tilted magnetic fields^28^ or by designing different sample geometries. Combined with conventional device fabrication techniques, such measurements are more feasible with film samples, and may provide an experimental answer also to the discussion, regarding the additional phase acquisition caused by bulk tunneling of the Weyl orbit^4,5,8,12–14^. Fabrication of hetero-interfaces for proximitizing the surface Fermi-arcs with ferromagnets and superconductors, as has been in progress in different contexts for Cd_3_As_2_ (refs. ^29–32^), could also lead to novel transport physics and functionalities. In this respect, our work revealing the unique distribution of Weyl orbits in the quantized transport paves the way for further exploring the potential of exotic surface transport phenomena in topological semimetals.

## Methods

### Film growth and device fabrication

The 75 nm thick (Cd_1−*x*_Zn_*x*_)_3_As_2_ film was grown on a SrTiO_3_ (100) single-crystalline substrate by the combination of pulsed laser deposition and solid-phase epitaxy^15,22,23^. By introducing a shadow mask during the deposition, the film was patterned into a Hall bar shape with a channel width of 60 μm. The deposited film was further capped by MgO (5 nm)/Si_3_N_4_ (200 nm) to prevent its evaporation and oxidization in the subsequent thermal annealing at 600 °C in air. For the dual-gate measurements, the top-gate configuration was fabricated by first etching the topmost Si_3_N_4_ capping layer down to 10 nm by ion milling, then depositing a 30 nm thick Al_2_O_3_ dielectric layer by atomic layer deposition, and lastly depositing a 50 nm thick Au layer as top-gate electrode. The back-gate configuration, on the other hand, was fabricated by simply using the SrTiO_3_ substrate as back-gate dielectric.

### Low-temperature transport measurement

The low-temperature measurements up to 9 T were performed in a physical property measurement system (PPMS, Quantum Design). The electric current was kept along the [100] axis of the SrTiO_3_ substrate, corresponding to the [$11\bar{1}$] or [$1\bar{1}0$] axis of the (Cd_1−*x*_Zn_*x*_)_3_As_2_ film which has 90° in-plane rotated domains formed by interfacial stacking of the (Cd_1−*x*_Zn_*x*_)_3_As_2_ hexagonal lattice on top of the SrTiO_3_ tetragonal lattice^22^. All of the transport measurements with gate bias were performed using a conventional lock-in technique. The excitation current was kept constant at 0.5 μA with the frequency set to 13 Hz. The *V*_TG_–*V*_BG_ (*V*_TG_–*B*) mapping was obtained by scanning *V*_TG_ with increasing *V*_BG_ (*B*) stepwise. The conversion of the *V*_TG_ axis to the sheet density *n*_2D_ axis is performed by referring to the low-field Hall measurement at each *V*_TG_.

## Supplementary information

Supplementary Information

## Data Availability

The data supporting the plots within the paper and its Supplementary Information file are available from the corresponding author upon reasonable request.
