# Supplementary material for: Intrinsic coupling between spatially-separated surface Fermi-arcs in Weyl orbit quantum Hall states
Source: Nat Commun. 2021 May 6;12:2572. doi: 10.1038/s41467-021-22904-8 (PMC8102497; doi:10.1038/s41467-021-22904-8)
Supplement: Supplementary file 1 — Supplementary Information [file 41467_2021_22904_MOESM1_ESM.pdf]

Intrinsic coupling between spatially-separated surface Fermi-arcs in Weyl orbit quantum Hall states

Shinichi Nishihaya[1], Masaki Uchida[1,2,3,*] Yusuke Nakazawa[1], Markus Kriener[4], Yasujiro Taguchi[4], & Masashi Kawasaki[1,4]

[1]*Department of Applied Physics and Quantum-Phase Electronics Center (QPEC), University of Tokyo, Tokyo 113-8656, Japan*

[2]*Precursory Research for Embryonic Science and Technology (PRESTO), Japan Science and Technology Agency (JST), Tokyo 102-0076, Japan*

[3]*Department of Physics, Tokyo Institute of Technology, Tokyo 152-8550, Japan*

[4]*RIKEN Center for Emergent Matter Science (CEMS), Wako 351-0198, Japan*

Supplementary Note 1 | Top-gate modulation of quantum transport at various back-gate voltages

Supplementary Fig. 1 presents top-gate voltage V_{TG} scans of transverse magnetoresistance R_{xx} and Hall resistance R_{yx} with various back-gate voltages. As V_{BG} is increased positively, the surface quantum Hall states become more unclear. One explanation for this observation may be that the bulk carrier density is not low enough to develop a clear surface dominated conduction. Another possible reason is the breaking of the Weyl orbit due to too strong asymmetry induced by the electric fields. The field configuration with $V_{\mathrm{TG}}< 0$ and $V_{\mathrm{BG}}> 0$ is such that the field-induced Weyl node shift is in the opposite direction between the top and bottom surfaces (see also Supplementary Note 5), which hinders the bulk tunneling part of the Weyl orbit. The decay of the quantum Hall signature may reflect such a deformation process of the Weyl orbits by the electric fields.

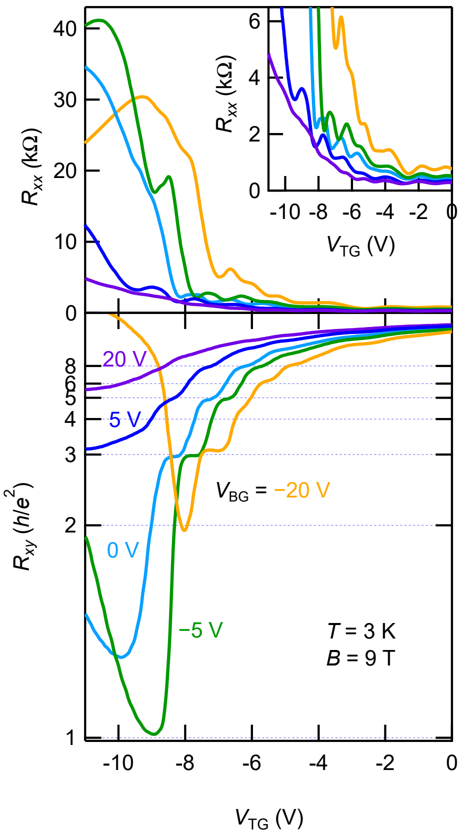

Supplementary Fig. 1 | Top-gate modulation of quantum transport at various back-gate voltages. Inset of the upper panel displays the magnified view of the quantum oscillations in the lower-resistance region.

Supplementary Note 2 | Two-carrier transport around the charge neutrality point

Supplementary Fig. 2 presents magneto-transport data measured at $V_{\mathrm{TG}}= -10$ V and $V_{\mathrm{BG}}= -20$ V which is after the system has crossed the charge neutrality point from the n-type to the p-type region as shown in Fig. 2c in the main text. To extract the density n_e (n_h) and mobility μ_e (μ_h) of the electrons (holes), the R_{yx} curve is fitted to the following conventional two-carrier transport model,

$$R_{yx}(B) = \frac{-{\mu_{\mathrm{e}}}^2 n_{\mathrm{e}} + {\mu_{\mathrm{h}}}^2 n_{\mathrm{h}} + (\mu_{\mathrm{e}}\mu_{\mathrm{h}}B)^2(-n_{\mathrm{e}} + n_{\mathrm{h}})}{e[(\mu_{\mathrm{e}}n_{\mathrm{e}} + \mu_{\mathrm{h}}n_{\mathrm{h}})^2 + (\mu_{\mathrm{e}}\mu_{\mathrm{h}}B)^2(-n_{\mathrm{e}} + n_{\mathrm{h}})^2]}B, R_{xx}(0) = \frac{1}{e(\mu_h n_h + \mu_e n_e)}. \quad (1)$$

The much lower mobility of the hole carriers is consistent with previous reports and is ascribed to the larger mass of the valence band[1,2].

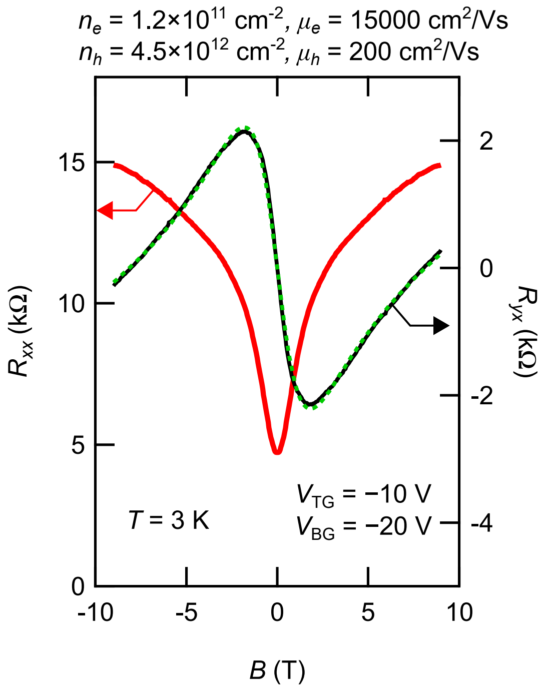

Supplementary Fig. 2 | Two-carrier transport around the charge neutrality point. Hall measurements with V_{TG} and V_{BG} set to -10 V and -20 V, respectively. The dashed line overlaid on the R_{yx} curve is the fitting result to the two-carrier model, indicating low-mobility holes as majority carriers.

Supplementary Note 3 | Bulk quantum oscillations in the longitudinal magnetic field

Supplementary Fig. 3 presents the B-V_{TG} mapping of longtudinal magnetoresisntance measured at V_{BG} = 0 V and -20 V. The data shown in the lower panels of Fig. 2b and 2c in the main text correspond to line cuts at $B = 9$ T in Supplementary Fig. 3a. In both the mappings in Supplementary Figs. 3a and 3b, bulk quantum oscillations reflecting the Landau level splitting of the three-dimensional bulk state are clearly observed. The system reaches the quantum limit under sufficiently large V_{TG} (< 0) after the appearance of a bulk oscillation peak assigned to the Landau index $N = 1$.

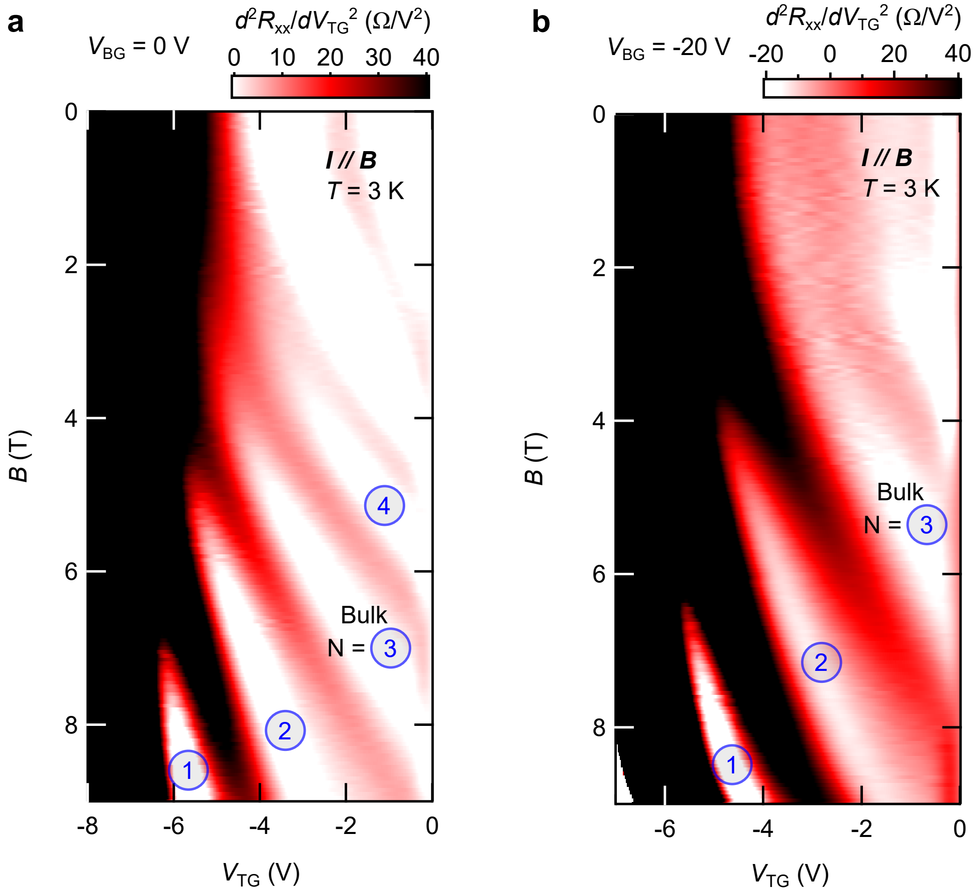

Supplementary Fig. 3 | Mapping of bulk quantum oscillations as a function of field and top-gate voltage. a,**b**, The back-gate voltage V_{BG} is set to 0 V (**a**) and -20 V (**b**).

Supplementary Note 4 | Surface quantum Hall states measured with different electric field configurations

Supplementary Figs. 4 and 5 present mappings of the surface quantum Hall states as a function of magnetic field B and sheet electron density n_{2D} measured at $V_{\mathrm{BG}} = 0$ V and -20 V. The n_{2D} axis is converted to the quantum Hall filling factor ν according to $\nu = n_{\mathrm{2D}}h/eB$ (e is the elementary charge and h the Planck constant). Representative line cuts of R_{yx} and its first derivative are also shown as a function of ν in Supplementary Figs. 4b-e and 5b-e. In Supplementary Fig. 6, we compare the converted B-ν mappings measured at $V_{\mathrm{BG}} = 0$ V (same as Fig. 4a in the main text) and -20 V. Importantly, the two mappings exhibit different appearances of filling factors and plateau transitions between them. Application of electric fields not only modulates the carrier density of the surface orbits, but also induces a splitting of the doubly degenerate Dirac nodes into a pair of Weyl nodes due to inversion symmetry breaking (see also Supplementary Note 5). It has also been pointed out that the field effect modifies the band structure of the surface state, such as by inducing shift of the surface Dirac point and by changing the Fermi velocity[3]. In this respect, the difference between the mapping results in Supplementary Fig. 6 provides experimental evidence that the configuration of the electric fields (inset of Supplementary Fig. 6) is an important factor for details of the degeneracy lifting of the two Weyl orbits. While in this work VTG is always set to be negative in order to deplete the high bulk residual electron density and to access the surface quantum Hall regime, performing measurements with different gate configurations such as ($V_{\mathrm{TG}}> 0$, $V_{\mathrm{BG}}> 0$) and ($V_{\mathrm{TG}}> 0$, $V_{\mathrm{BG}}< 0$) by using lower electron density samples may provide

further insight in the degeneracy lifting mechanism of the Weyl orbits.

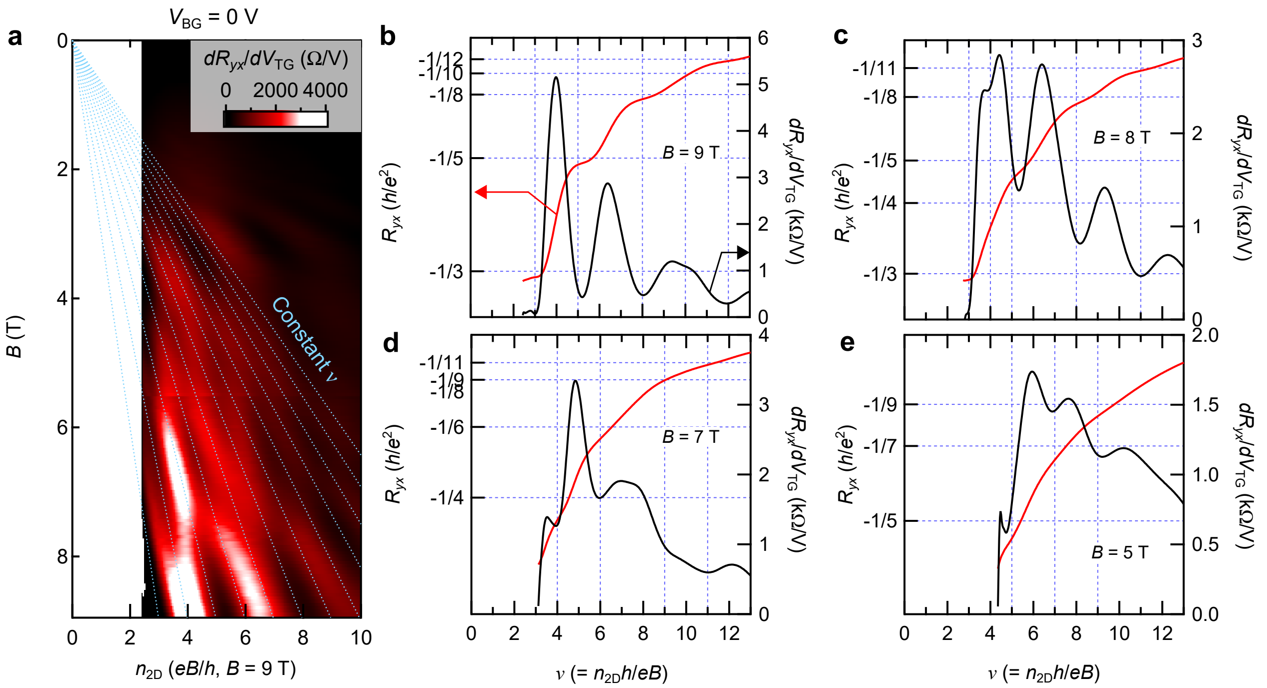

Supplementary Fig. 4 | Surface quantum Hall states measured at back-gate voltage V_{BG} = 0 V. **a**, Mapping of the first derivative of R_{yx} with respect to V_{TG} as a function of magnetic field B and sheet carrier density n_{2D}. **b-e**, R_{yx} (left) and its derivative (right) shown as a function of filling factor ν.

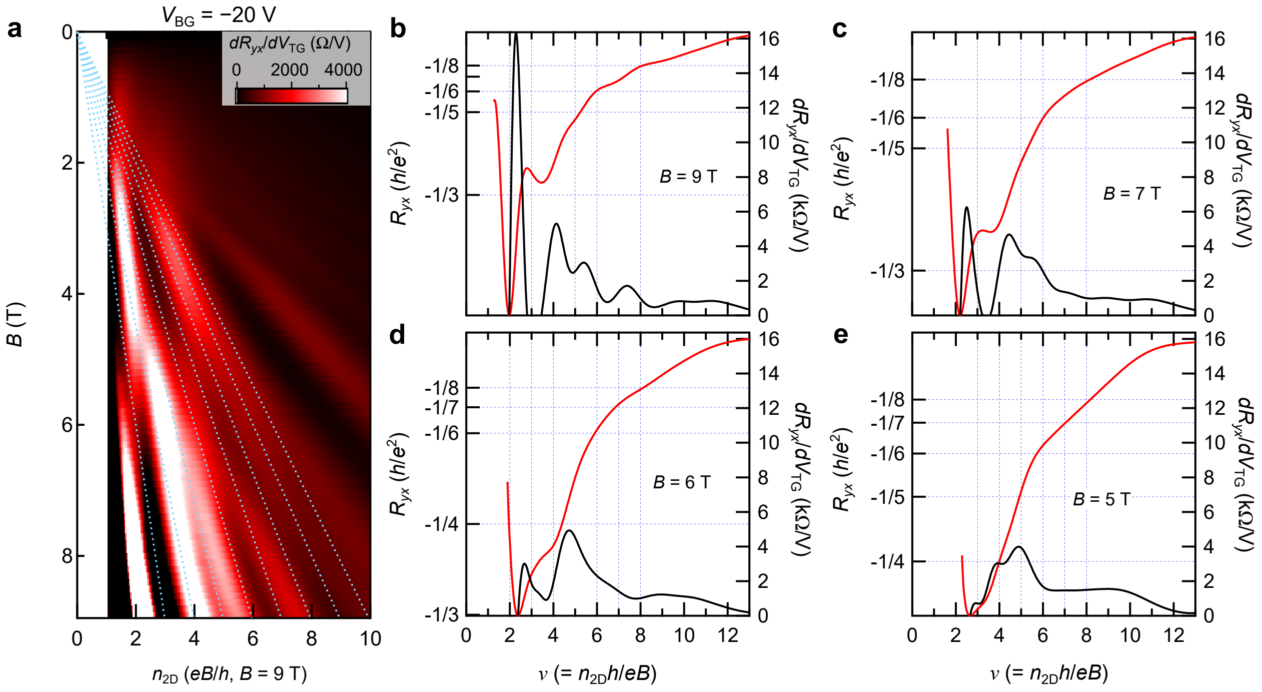

Supplementary Fig. 5 | Surface quantum Hall states measured at back-gate voltage V_{BG} = -20 V. a, Mapping of the first derivative of R_{yx} with respect to V_{TG} as a function of magnetic field B and sheet carrier density n_{2D}. **b-e**, R_{yx} (left) and its derivative (right) shown as a function of filling factor ν.

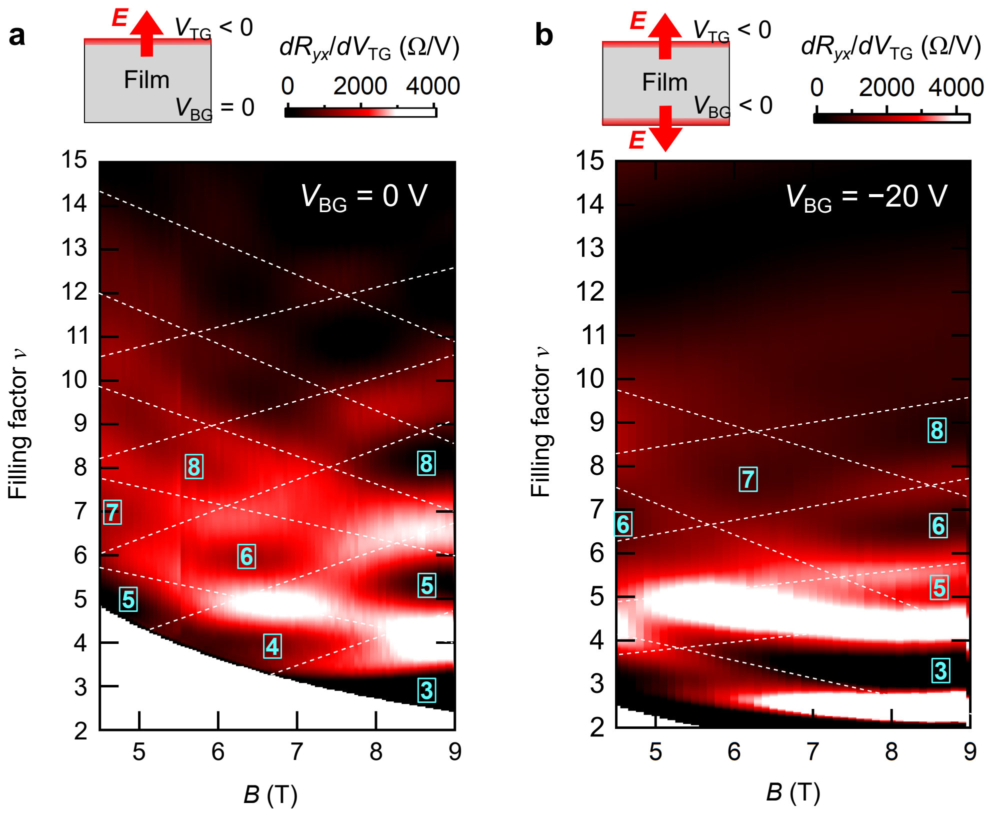

Supplementary Fig. 6 | Comparison of plateau transitions measured with different electric field configurations. a,**b**, Mapping of the first derivative of R_{yx} with respect to V_{TG} as a function of magnetic field B and filling factor ν for the cases $V_{\mathrm{BG}} = 0$ V (**a**) and $V_{\mathrm{BG}} = -20$ V (**b**). The dashed lines are guides for eyes. The schematic on top of each figure illustrates the electric field configuration.

Supplementary Note 5 | Degeneracy lifting of the paired Weyl orbits by external fields

Mapping of the surface quantum Hall states as a function of magnetic field and gate-voltages reveal not only the Weyl orbit type coupling of the top and bottom surfaces, but also the signature of degeneracy lifting of the two Weyl orbits, resulting in complex plateau transitions depending on the external fields. Either breaking time-reversal symmetry by the magnetic field B or breaking inversion symmetry by the electric field E can induce a phase transition from a Dirac semimetal (DSM) to a Weyl semimetal (WSM). Both the E-driven and B-driven splitting of the DSM phase result from the separation of the Weyl nodes of opposite chirality in energy and momentum directions[3–5]. The change of bulk node separation directly modulates the Fermi surface area enclosed by the Weyl orbit as shown in Fig. 4b in the main text, thus leading to the degeneracy lifting of the two Weyl orbits of opposite chirality.

In the case of B-driven splitting, the bulk Zeeman splitting induces the Weyl node shift, where its orbital independent part is responsible for the shift in the energy direction, and its orbital dependent part for the shift in the momentum direction[4]. Because of the large g-factor of Cd_3As_2 conduction band especially in the vicinity of the band bottom[6,7], the B-driven splitting effect on the Weyl orbits is not negligible. For incorporating this effect in the simulation of the Landau fan diagram presented in Fig. 4c, we assume a Zeeman-like splitting between the two Weyl orbits by introducing an effective g-factor g^*.

For the E-driven splitting, at a given field direction, the node shift along the energy and

momentum axes is expected to be opposite depending on the chirality and also the surface position (top or bottom). Moreover, it has been proposed that the application of the electric field directly modulates the surface dispersion by inducing a renormalization of the Fermi velocity, which also takes place asymmetrically between opposite chirality and surface position[3]. Because this E-driven effect at certain V_{TG}-V_{BG} configuration does not appear explicitly in the Landau fan diagram which is plotted as a function of magnetic field, we incorporate this effect by introducing an energy offset E_{offset} between the Landau levels of the two Weyl orbits, and also by modifying the band parameters such as the mass m and Fermi velocity v_{F}.

Based on the Landau level splitting of a 2D Dirac dispersion, and by introducing the B-driven and E-driven splitting effects between the two Weyl orbits, we show that multiple crossings of the Landau levels, which qualitatively account for our experimental observation presented in Fig. 4a, can be simulated. Below we provide the details of the model used.

In the presence of non-trivial Z_2 invariant resulting from the band inversion, the two surface Fermi-arcs of DSM generally cross each other to form a 2D Dirac dispersion. Similar to the topological insulator case, the surface Dirac dispersion is expected to host a single Dirac point residing at the center of the Brillouin zone[8,9]. The Landau level energy of such 2D Dirac system placed in an out-of-plane magnetic field can be obtained by using the conventional model Hamiltonian for the surface state of a topological insulator[10],

$$H = v_F(\sigma_x \Pi_y - \sigma_y \Pi_x) + \frac{1}{2m}(\Pi_x^2 + \Pi_y^2) + \frac{1}{2}\gamma g^* \mu_B B \sigma_z. \quad (2)$$

Here, e the elementary charge, and v_{F} the Fermi velocity. $\mathbf{\Pi}$ is the canonical momentum after the Peierls substitution $\mathbf{\Pi} = \hbar \boldsymbol{k} + e\boldsymbol{A}$, where $\boldsymbol{k}$ denotes momentum and $\boldsymbol{A}$ vector potential. $\boldsymbol{\sigma}$ refers to the Pauli matrices. Here we have introduced the Zeeman term $\frac{1}{2}\gamma g^* \mu_B B \sigma_z$ to effectively describe the B-driven splitting of the two Weyl orbits. The Zeeman term has opposite sign for opposite chirality $\gamma = \pm 1$. We have also included a parabolic term $\frac{1}{2m}(\Pi_x^2 + \Pi_y^2)$ in order to take into account a possible contribution from the finite curvature of the surface dispersion. Then, the Landau level energies are given as follows[10],

$$E_{N \neq 0} = \hbar\omega_c N + sgn(N)\sqrt{2\hbar v_F^2 e|N|B + (\frac{1}{2}\hbar\omega_c - \frac{1}{2}\gamma g^* \mu_B B)^2} \quad (3)$$

$$E_{N=0} = \frac{1}{2}\hbar\omega_c - \frac{1}{2}\gamma g^* \mu_B B. \quad (4)$$

Here, N is the Landau index ($N = 0, \pm 1, \pm 2, \cdots$), and the cyclotron energy $\hbar\omega_c$ is given by $\hbar\omega_c = \hbar eB/m$. Only assuming the effective Zeeman term, however, the multiple crossings of the Landau levels between the two Weyl orbits in Fig. 4a do not occur as shown in Supplementary Fig. 7a. To account for such a level crossing pattern, it is necessary to consider an energy offset E_{offset}, which corresponds to the contribution from the E-driven splitting of the Weyl orbits. Therefore, Fig. 4c in the main text is obtained by further including a finite energy offset between the two sets of Landau levels (Supplementary Fig. 7b). We have also chosen suitable values for the masses (m_1, m_2) and the Fermi velocities ($v_{\mathrm{F},1}$,$v_{\mathrm{F},2}$) of the two Weyl orbits as well as the effective g^* so that the crossings of the Landau levels occur in a similar way as observed experimentally in Fig. 4a.

While the simulation in Supplementary Fig. 7b accounts for the experimental case of Sup-

plementary Fig. 6a ($V_{\rm TG}< 0$, $V_{\rm BG}= 0$), turning on $V_{\rm BG}< 0$ leads to the different Landau level crossing pattern as presented in Supplementary Fig. 6b. This can be ascribed to the increase of Eoffset between the two Weyl orbit Landau levels, as the surface bands of opposite chirality on the bottom surface start to have energy splitting contribution in addition to that of the top surface. From this viewpoint, the $V_{\rm BG}$-induced level crossing denoted by dashed lines in Fig. 3b in the main text can be qualitatively explained by the crossing between the red $N = 5$ and the blue $N = 3$ Landau levels in Supplementary Fig. 7b, as the $E_{\rm offset}$ term increases upon the application of $V_{\rm BG}< 0$.

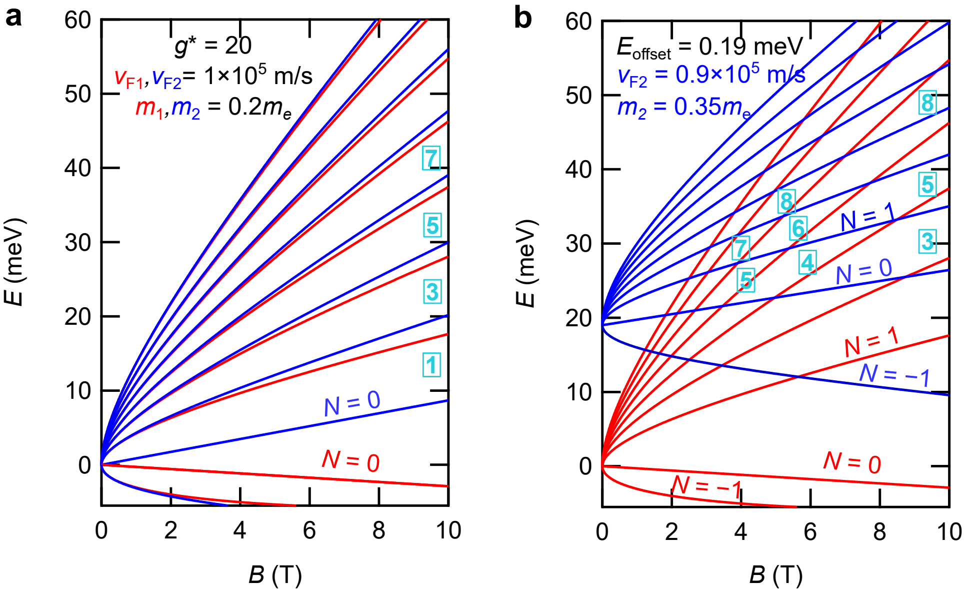

Supplementary Fig. 7 | Simulation of Landau fan diagram. a,b, Simulation results without energy offset $E_{\rm offset}$ (**a**) and with $E_{\rm offset}$ (**b**) between the two sets of Landau levels. The masses (m_1, m_2) and Fermi velocities ($v_{\rm F,1}$, $v_{\rm F,2}$) of the two Weyl orbits are also modified in **b** so that the Landau level crossings well account for the experimental result in Fig. 4a.

Supplementary References

1. Nishihaya, S. *et al*. Gate-tuned quantum Hall states in Dirac semimetal $(Cd_{1-x}Zn_x)_3As_2$. *Sci. Adv.* **4,** eaar5668 (2018).

2. Liu, Y. *et al*. Gate-tunable quantum oscillations in ambipolar Cd_3As_2 thin films, NPG Asia Mater. **7,** e221 (2015).

3. Baba, Y., Díaz-Fernández, Á., Díaz, E., Domínguez-Adame, F. & Molina, R. A. Electric field manipulation of surface states in topological semimetals. *Phys. Rev. B* **100,** 165105 (2019).

4. Wang, Z. *et al*. Dirac semimetal and topological phase transitions in A_3Bi (A = Na, K, Rb). *Phys. Rev. B* **85,** 195320 (2012).

5. Gorbar, E. V., Miransky, V. A. & Shovkovy, I. A. Engineering Weyl nodes in Dirac semimetals by a magnetic field. *Phys. Rev. B* **88,** 165105 (2013).

6. Wallace, P. R. Electronic g-factor in Cd_3As_2. *Phys. Stat. Sol. (b)* **92,** 49-55 (1979).

7. Jeon, S. *et al*. Landau quantization and quasiparticle interference in the three-dimensional Dirac semimetal Cd_3As_2. *Nat. Mater.* **13,** 851-856 (2014).

8. Yang, B.-J. & Nagaosa, N. Classification of stable three-dimensional Dirac semimetals with nontrivial topology. *Nat. Commun.* **5,** 4898 (2014).

9. Kargarian, M., Randeria, M. & Lu, Y.-M. Are the surface Fermi arcs in Dirac semimetals topologically protected? *Proc. Natl. Acad. Sci. USA* **113,** 8648-8652 (2016).

10. Reynoso, A., Usaj, G., Sánchez, M. J. & Balseiro, C. A. Theory of edge states in systems with Rashba spin-orbit coupling. Phys. Rev. B **70,** 235344 (2004).
